# Chlorogenic Acid Decreases Malignant Characteristics of Hepatocellular Carcinoma Cells by Inhibiting DNMT1 Expression

**DOI:** 10.3389/fphar.2020.00867

**Published:** 2020-06-10

**Authors:** Yao Liu, Ying Feng, Yuxin Li, Ying Hu, Qun Zhang, Yunyi Huang, Ke Shi, Chongping Ran, Jie Hou, Guiqin Zhou, Xianbo Wang

**Affiliations:** ^1^Center of Integrative Medicine, Beijing Ditan Hospital, Capital Medical University, Beijing, China; ^2^Department of Gastroenterology, Dongzhimen Hospital, Beijing University of Chinese Medicine, Beijing, China

**Keywords:** chlorogenic acid, DNMT1, hepatocellular carcinoma, proliferation, migration

## Abstract

**Background:**

Hepatocellular carcinoma (HCC) is the most common malignant tumor of the adult liver, exhibiting rapid progression and poor prognosis. Chlorogenic acid (CGA), a polyphenol, has several biological activities, including the suppression of liver cancer cell invasion and metastasis. Increased levels or alterations in the function of DNMT1 are associated with the inactivation of tumor suppressor genes. However, the CGA-affected DNMT1 expression mediated mechanism is still unclear.

**Methods:**

The human hepatocellular carcinoma (HCC) HepG2 cells were treated with a positive control drug (5-AZA) or varying doses of CGA. DNA methyltransferase 1 (DNMT1) protein levels and other relevant proteins were evaluated using Western blotting and immunocytochemistry. Cell-cycle analysis was performed by flow cytometry-based PI staining, and cell viability was assessed using 3-(4,5-dimethylthiazol-2-yl)-2,5-diphenyltetrazolium bromide (MTT) assay. The transwell invasion and wound healing assays were used to evaluate cell migration and invasion. In vivo proliferation of the HCC cells was detected. We investigated the expression of DNMT1, p53, p21, p-ERK, MMP-2, and MMP-9 in tumors using immunohistochemical analysis.

**Results:**

Our results showed that CGA inhibited the proliferation, colony formation, invasion, and metastasis of HepG2 cells both *in vitro* and *in vivo* by down-regulating DNMT1 protein expression, which enhanced p53 and p21 activity, and resulting in a significant reduction in cell proliferation and metastasis. Moreover, CGA inactivated ERK1/2 and reduced MMP-2 and MMP-9 expression in HepG2 cells.

**Conclusions:**

CGA can suppress liver cancer cell proliferation, invasion, and metastasis through several pathways. CGA could serve as a candidate chemopreventive agent for HCC.

## Introduction

Hepatocellular carcinoma (HCC) is the most common adult liver malignancy exhibiting rapid progression and poor prognosis(Shimada et al., 1996; Ma et al., 2014). Many signaling pathways participate in the development of HCC. In addition to several possibilities causing HCC development, studies have implicated the accumulation of genetic abnormalities (e.g., epigenetic changes and gene amplification, as well as chromosomal variations) in triggering the development of HCC (Nishida and Goel, 2011). Epigenetic alterations are heritable variations that are not attributed to DNA sequence variations. DNA methylation is an important research topic in epigenetics.

DNA methylation is the addition of a methyl group to a cytosine (C) residue assisted by an enzyme, DNA methyltransferase 1 (DNMT1); it is the most prevalent epigenetic modification (Jones and Baylin, 2002; Hegi et al., 2009). Gene promoter hypermethylation has been considered as a vital mechanism to inhibit the expression of genes involved in tumor development. In sporadic cancers, roughly half the tumor-suppressor genes are reported to be inactivated by epigenetic rather than genetic mechanisms (Xu et al., 2018). Protein-protein interactions (PPIs) were used to identify the interactions between DNMT1 and tumor progression-related proteins (https://string-db.org/). The results showed a link between DNMT1 and p53.

p53 is a tumor-suppressor protein. Under normal conditions, p53 is turned off and activated during stress when cells divide and proliferate uncontrollably (Vogelstein et al., 2000; Harris and Levine, 2005). As cell growth is out of control, p53 induces p21 expression, which results in cell-cycle arrest (Faria et al., 2007; Georgakilas et al., 2017). Previous studies have shown a relationship between the methylation of CpG dinucleotide located in the p53 promoter region with low p53 expression levels, leading to tumor progression and growth.

Since tumor-suppressor gene promoter hypermethylation is considered as one of the principal factors facilitating tumor progression, demethylation drugs have become the primary research focus for molecularly targeted therapy (Sajadian et al., 2016). Many different *in vitro* studies showed that 5-azacytidine (5-AZA), a potent DNA methyltransferase inhibitor (DNMTi), triggers the re-expression of silenced genes, alters the expression of genes participating in tumor suppression (Christman, 2002), and is used as a positive control drug. Chlorogenic acid (CGA), a polyphenol, is an ester in which the acid (part of the caffeic acid) binds to the hydroxyl group at 5′ of the quinic acid (5′-coffee-derived quinic acid). Epidemiological studies suggested that CGA has antioxidant, anti-inflammatory, antiviral, and anticancer properties, and other biological characteristics (Shi et al., 2009; Xu et al., 2010; Yun et al., 2012; Zhao et al., 2012; Ji et al., 2013; Shi et al., 2013). A recent study showed that CGA could prevent HCC progression by inactivating ERK1/2 and suppressing MMP-2 and MMP-9 expressions (Yan et al., 2017). Furthermore, by inhibiting the activity of the anti-apoptotic proteins Bcl2 and Bcl-xL and activating the pro-apoptotic proteins annexin V, Bax, and caspase 3/7, CGA promoted regorafenib’s apoptotic effect (Refolo et al., 2018). However, the CGA-affected DNMT1 expression-mediated mechanisms are still unclear.

In this study, we evaluated the direct effect of CGA on the HCC cells. CGA inhibited *in vitro* HepG2 cell proliferation by inactivating DNMT1 and activating P53 and increasing the expression of p21. In addition, CGA inactivated ERK1/2 and reduced the expression of MMP-2 and MMP-9 in HepG2 cells. Based on the data mentioned above, CGA exhibits anti-proliferative activity and could be a potential therapeutic agent for the treatment of HCC.

## Materials and Methods

### Materials

CGA was provided by J&K Scientific. Ltd (Beijing, China), was dissolved in sterile H_2_O, the solution filtered using a 0.22-μm filter, incubated at −20°C, and diluted with the cell culture medium. 5-Azacytidine was obtained from Melone Pharmaceutical Co. Ltd (Dalian, China), dissolved in sterile Dimethyl Sulfoxide (DMSO, Sigma), and incubated at −20°C. The cells in the CGA group were treated for 48 h with graded doses of CGA (0, 250, 500, and 1000 μM). The cells in the 5 -AZA group were treated for 48 h with varying doses of 5-AZA (0, 1, 5, and 10 μM), and vehicle DMSO was present at an equal concentration in the control group (CON). The antibodies for DNMT1, p21, MMP2, MMP9, and GAPDH were procured from Cell Signaling Technology, Inc. (Danvers, MA, USA). The antibodies against p53 were obtained from Proteintech Group, Inc. (Wuhan, Hubei, China).

### Cell Culture

The HCC cell lines (HuH-7, HepG2, MHCC97H, and MHCC97L) were provided by the China Infrastructure of Cell Line Resources. Cells were cultured with Dulbecco’s modified Eagle’s medium (DMEM) containing 1% glutamine, 1% penicillin/streptomycin, and 10% fetal bovine serum (FBS; Hyclone, USA). Cells were incubated at 37°C with 5% CO_2_ and were passaged once every 2–3 days, and cells in the mid-log phase were used for all experiments.

### Cell Viability Assay

Cell viability was assessed with the 3-(4,5-dimethylthiazol-2-yl)-2,5-diphenyltetrazolium bromide (MTT) method. HepG2 cells were seeded in 96-well plates (4×10^3^ cells/well) and incubated for 96 h at 37°C with 5% CO2 in the presence of increasing concentrations of CGA and 5-AZA. At the end of 96 h, 20 μl of MTT solution (5 mg/ml) was added to each well and incubated for 4 h at 37°C. Next, the optical density was measured at 490 nm, and for normalization of the number of live cells, the absorbance values of cells dissolved in 150 μl of DMSO were used.

### Colony Formation Assays

We conducted colony formation assays for 48 h with 500 cells plated in six-well plates from each group treated with a fixed dose of 5-AZA (5 μM) and varying doses of CGA (250, 500, and 1000 μM). After 7 days of incubation, each well was washed with PBS and stained with crystal violet. The colonies were counted using a Leica DM6000B microscope with 10 × 0.25 NA objective lens (Leica, Wetzlar, Germany).

### Cell Cycle Analysis

Cells pretreated for 48 h with 5-AZA (5 μM), and different doses of CGA (250, 500, and 1000 μM) were harvested by trypsinization, and 1×10^6^ cells were used for analysis. The cells were washed twice with PBS and fixed in 75% ethanol at 4°C overnight. The cells were stained with propidium iodide for 30 min in the dark at 37°C following the manufacturer’s instructions (BD, USA). Later, cells were collected, and cell-cycle analysis conducted using a ﬂow cytometer (BD), and the histograms were analyzed using the ModFit software (Becton-Dickinson, USA).

### Transwell Invasion Assay

For the transwell chamber assays, we used a filter membrane with an 8 μm pore size, pre-coated with Matrigel (BD Biosciences, CA, USA) for invasion assays, while ECM was omitted for performing cell migration assays (Costar, NY, USA). We added 600 μl of complete medium (DMEM, 10% FBS) to the lower chamber. Later, diluted HepG2 (5 × 10^5^/ml) cells and 200 μl of the suspensions of CGA (250, 500, and 1000 μM)-, 5-AZA (5 μM)-pretreated cells and the vehicle DMSO control group (CON) were added to the upper chamber. For invasion assays, HepG2 cells were incubated for 24 and 48 h at 37°C in a humidified incubator with 5% CO_2_. The non-migratory cells over the top of the filter were removed, and the invaded cells were kept stationary using anhydrous ethanol and stained using crystal violet. Images were captured at 100× magnification and the number of invaded cells was counted in five image fields. No fewer than five random microscopic fields were observed using Leica DM6000B microscope with a 10 × 0.25 NA objective lens (Leica, Wetzlar, Germany).

### Wound Healing Assay

Cells pretreated with CGA (250, 500, and 1000 μM) were seeded in six-well plates, and when cells reached 90% confluence, the cells were scratched using a sterile pipette tip (10 μl). The scratched cells were washed with PBS and imaged using a Leica DM6000B microscope with 10 × 0.25 NA objective lens (Leica, Wetzlar, Germany). Later, 10% FBS in DMEM was added to cells for 48 h, and images of the wound were taken using the Image-Pro Plus (Media Cybernetics, USA), for measuring the scratch area.

### Western Blot Analysis

Cells were lysed in RIPA buffer supplemented with protease inhibitors. The protein concentrations were measured using the BCA Protein Assay kit (Pierce). Equal amounts of each protein sample (20 μg) were added to the sample wells and electrophoresed on 10% SDS-polyacrylamide gels. The separated proteins were transferred to polyvinylidene diﬂuoride membranes. The blotted membranes were blocked for 1 h with 5% skim milk, followed by incubation with anti-rabbit antibodies against DNMT1 (CST, 1:500 dilution), p53 (Proteintech, 1:500 dilution), p21^waf/Cip1^ (CST, 1:500 dilution), MMP2 (CST, 1:500 dilution), MMP9 (CST, 1:500 dilution), and GAPDH (CST, 1:1000 dilution). Horseradish peroxidase-conjugated IgG secondary antibodies were used to visualize the immunoreactive bands with optimized chemiluminescence, and the band intensity was measured by the Alpha View software (Protein Simple, USA).

### Immunocytochemistry Staining

For immunocytochemistry, HepG2 cells pretreated with CGA (500 μM) and 5-AZA (5 μM) for 48 h on glass coverslips were washed three times with phosphate-buffered saline, fixed for 30 min in absolute alcohol, followed by immunocytochemical staining for DNMT1 (1:500 dilution, CST), p53 (1:500 dilution, Proteintech), and p21 (1:500 dilution, CST). All steps were performed following the immunohistochemistry kit instructions. Diaminobenzidine (DAB) was used for color development. No fewer than five random microscopic fields were observed under Leica DM6000B microscope (Leica, Wetzlar, Germany), and the degree of immunostaining was measured using the Image-Pro Plus (Media Cybernetics, USA).

### Subcutaneous Xenograft Nude Mouse Models

The animal center at the Beijing Vital River provided nude mice (BALB/c-A, 4 weeks old, male); they were housed under specific pathogen-free conditions. The mice were given free access to aseptic water and food. Before the experiment, the nude mice were acclimatized for at least seven days. Then, HepG2 cells (5×10^6^ cells) were injected subcutaneously into the right flank of the mice. Tumors were measured using calipers every 5 days for 25 days, and the tumor size was calculated using the formula: V = 1/2 ab^2^, where “a” is the maximum tumor diameter and “b” is the minimum tumor diameter. When the subcutaneous tumors of the nude mice reached 100 to 200 mm^3^, the mice were divided randomly into four groups and received an intraperitoneal injection once per day (CON [normal saline], CGA [120 and 480 mg/kg], and 5-AZA [5 mg/kg). Mice bearing subcutaneous tumors were euthanized after 35 days. The tumor tissues were surgically resected, fixed in formalin, and embedded in paraffin. For the immunohistochemistry analysis, we used the paraffin-embedded tissues. The study protocol was approved by the Vital River Institutional Animal Care and Use Committee (Document Number: RSD-SOP-002-01, and Protocol Number: P2019037). All animal experiments were conducted in accordance with the recommendations in the Guide for the Care and Use of Laboratory Animals of the National Institutes of Health. Every effort was made to reduce the number of the animals used and minimize animal suffering.

### Immunohistochemistry

Formalin-fixed, paraffin-embedded tissue sections were de-paraffinized using washing steps with a graded range of alcohol solutions, and subsequent antigen retrieval and blocking with 5% BSA for 60 min. Tissue sections were incubated with antibodies against DNMT1 (1:400 dilution, Abcam), p53 (1:80 dilution, CST), p21 (1:50 dilution, CST), p-ERK (1:200 dilution, CST), MMP-2 (1:50 dilution, CST), and MMP-9 (1:50 dilution, CST) over-night at 4°C. After washing, the secondary goat anti-mouse IgG antibody (ZSGB-BIO, Beijing, China) was added and incubated for 1 h at room temperature. The tissue sections were stained with 3,3′-diaminobenzidine (ZSGB-BIO, Beijing, 121 China) and hematoxylin (Solarbio, Beijing, China). Images were captured using a ZEISS microscope (Carl Zeiss AG, Baden-Württemberg, Germany), and the degree of the immunostaining was measured using Image-Pro Plus (Media Cybernetics, USA).

### Statistical Analysis

Statistical analysis was by using the SPSS 20.0 (IBM, NY, USA) software, and results presented using the GraphPad software (GraphPad Software, CA, USA). The number of observations represents the categorical data. The mean ± standard deviation denoted the variables consistent with normal distributions. One-way ANOVA was used to analyze the differences in cell numbers, number of colonies, and tumor cell proliferation during various stages of the cell cycle among the experimental groups. A *p* value of < 0.05 was considered significant.

## Results

### Suppression of Cell Proliferation and Cell Cycle Progression in HepG2 Cells

**Figure 1:**
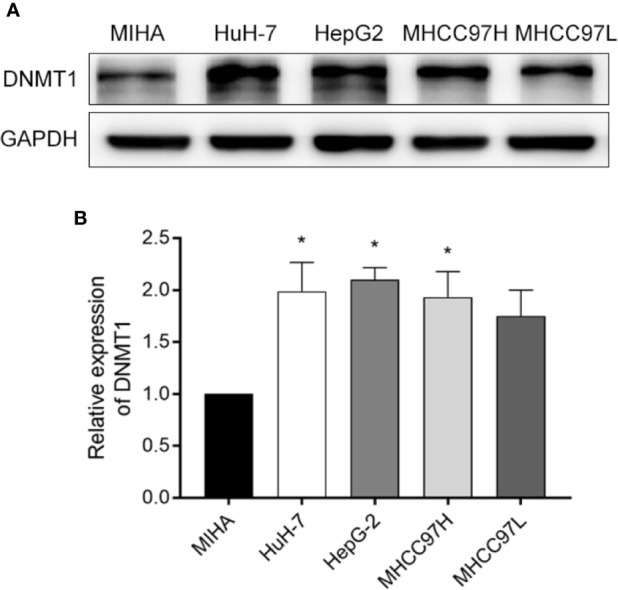
Screening cell lines. **(A**, **B)** A comparison of the Western blotting results of DNMT1 expression in HCC cell lines (HuH-7, HEPG2, MHCC97H, and MHCC97L), and a normal liver cell line (MIHA). *p < 0.05.

We confirmed DNMT1 expression in four commonly used HCC cell lines, HuH-7, HepG2, MHCC97H, MHCC97L, as well as MIHA, a normal liver cell line. The Western blot results showed a high expression of DNMT1 in HepG2 cells (**Figures 1A, B**).

**Figure 2:**
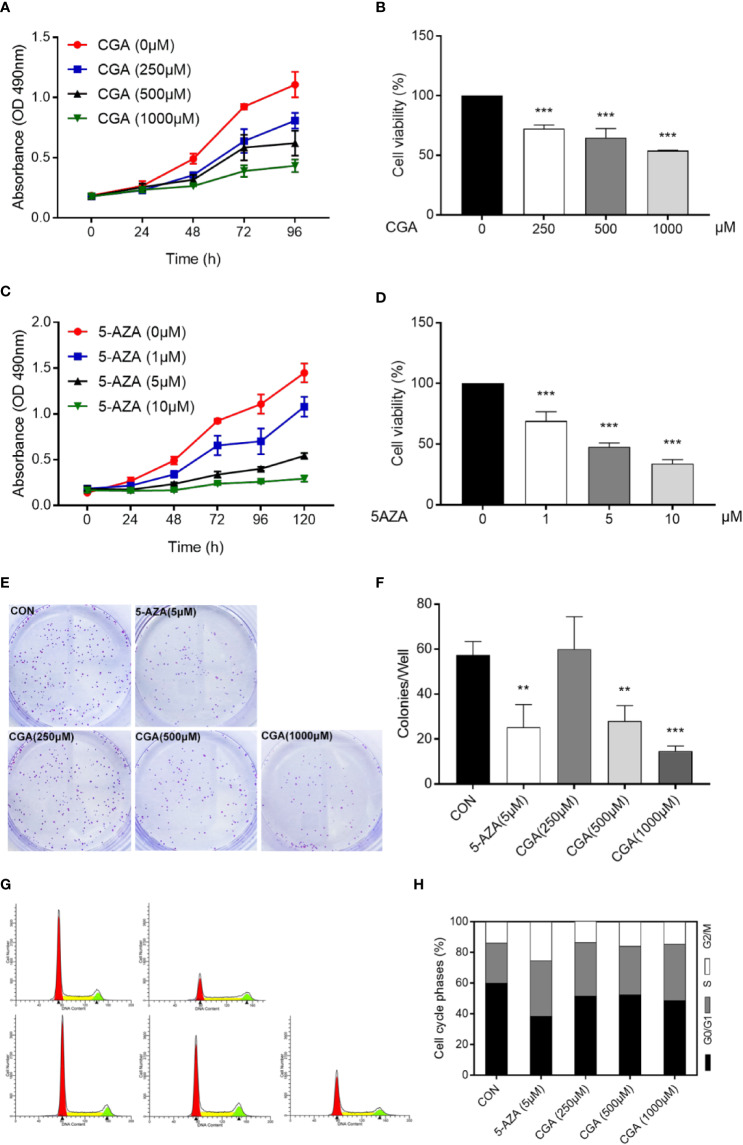
CGA and 5-AZA inhibit proliferation and trigger cell cycle arrest of human HepG2 cells. Human HepG2 cells were treated for 96 h with increasing concentration of CGA **(A, B)** and a fixed concentration of 5-AZA **(C, D)**. Subsequently, MTT assay assessed the cell viability as described in the Materials and Methods section. **(E, F)** HepG2 colony formation ability decreased remarkably after 48 h treatment with CGA and 5-AZA. **(G, H)** Cell cycle analysis shows increased S-phase arrest in cells from the CGA (1000 μM), and 5-AZA (5 μM) groups, compared to those from the CON group. **p < 0.01 and ***p < 0.001. CON, vehicle DMSO control group.

Cell proliferation was evaluated using MTT and colony formation assays. Cell proliferation was suppressed dose-dependently by CGA and 5-AZA, as shown by the MTT assay. At 500 and 1000 μM concentrations, CGA decreased the cell viability to 64.63%, and 53.88%, respectively; at 1 and 5 μM concentrations, 5-AZA decreased the cell viability by 69.10%, and 47.56%, respectively, at 48 h (*p* < 0.001, **Figures 2A–D**). Colony formation assay results showed that cells pretreated with CGA and 5-AZA exhibited fewer and smaller colonies than the control group, and with increasing CGA concentrations, the effect was more significant (**Figures 2E, F**).

Furthermore, cell cycle analysis was conducted to evaluate the CGA- and 5-AZA-mediated suppression of HepG2 cell viability. The data showed that both CGA and 5-AZA caused S-phase arrest in HepG2 cells (**Figures 2G, H**). These *in vitro* results suggest the CGA-mediated inhibition of HepG2 cell proliferation.

### CGA and 5-AZA Inhibit *In Vitro* Invasion and Migration of HCC Cells

**Figure 3:**
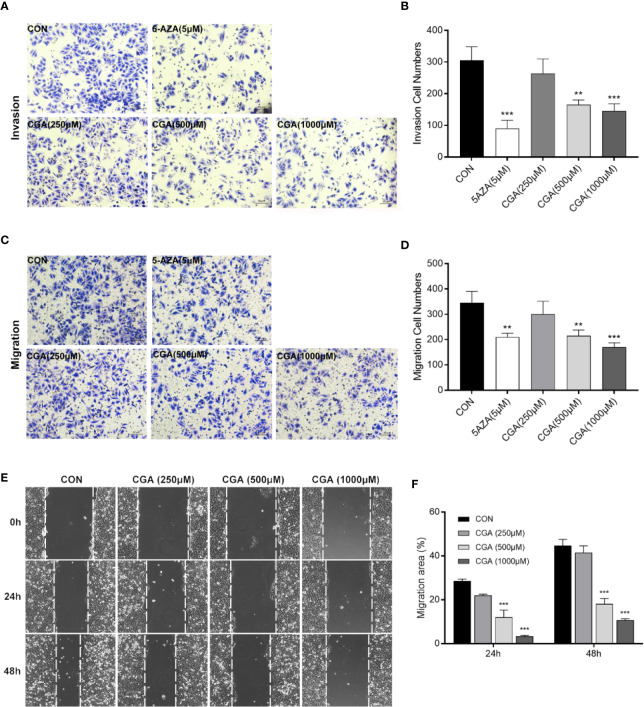
CGA and 5-AZA inhibit invasion and migration of HEPG2 cells. After treatment with CGA (250, 500, 1000 μM) or 5-AZA (5 μM), cell invasion **(A, B)** and migration **(C, D)** abilities were detected by transwell invasion assays, and the migration rate was measured by the wound healing assay **(E, F)** in HEPG2 cells. **p < 0.01, ***p < 0.001. CON, vehicle DMSO control group.

Rapid invasion and migration of HCC cells lead to disappointing liver cancer treatment outcomes. For investigating the *in vitro* effect of CGA and 5-AZA on HCC cell invasion and migration processes, cells were treated with CGA at doses of 0, 250, 500, and 1000 μM and 5-AZA at 5 μM. HepG2 cell invasion and migration abilities were analyzed in transwell chambers. CGA suppressed the invasion of HCC cells dose-dependently (**Figures 3A, B**), with significant inhibition by CGA at 500 and 1000 μM. At 1000 μM, the percent suppression of invasion was 52.5% at 48 h in HepG2 cells compared to the control group. CGA inhibited the migration of HepG2 cells (**Figures 3C, D**). In Boyden chamber assays without Matrigel, CGA caused considerable suppression of HepG2 cell migration by 50.7% compared to the control group at 24 h. In contrast, 5 μM 5-AZA reduced the invasion ability by 70.5% and suppressed migration by 39.1% compared to the cells in the control group at 48 and 24 h, respectively.

In addition, to characterize the effect of CGA on HepG2 cells’ migration, we performed wound healing assays. Treatment with CGA at doses of 500 and 1,000 μM significantly reduced the migratory ability of HepG2 in would healing assays over 24 and 48 h (**Figures 3E, F**). Altogether, based on the data mentioned above, CGA and 5-AZA can suppress HCC cells from metastasizing *in vitro*.

### CGA Suppresses DNMT1 and Up-Regulates p53 and p21

**Figure 4:**
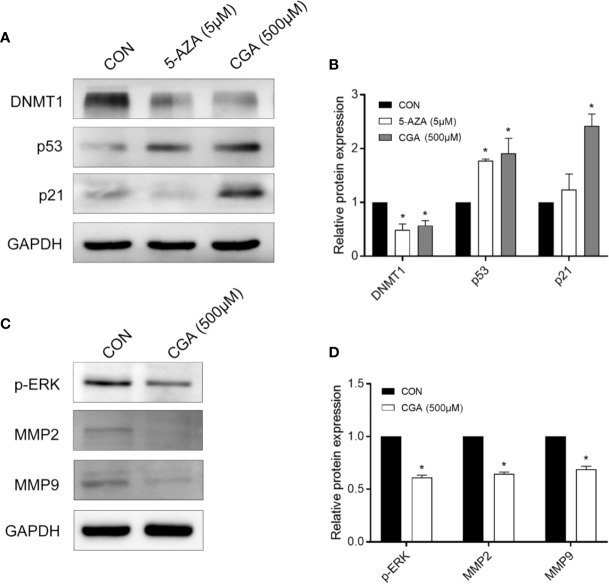
CGA and 5-AZA inhibit the expression of DNMT1 and up-regulate p53, p21waf/Cip1, to suppress HCC cell proliferation. **(A)** CGA inhibits the expression of ERK1/2 and MMP2/9 to suppress HCC growth. **(C)** Evaluation of the protein expression levels involved in the DNMT1/p53/p21 pathway and p-ERK/MMP2/MMP9 pathway. **(B, D)** The gray value statistics of protein DNMT1, p53, p21, p-ERK, MMP2, and MMP9. *p < 0.05. CON, vehicle DMSO control group.

P21 is a vital signal transducer for proliferation processes. Western blot assays were performed to confirm the effect of CGA on DNMT1 activity, p53, and p21, and the results show decreased levels of DNMT1 in cells after treatment with CGA or 5-AZA for 48 h. However, the levels of p53 and p21 increased as the DNMT1 levels decreased (**Figure 4A**). The protein levels in the cells from the CGA (500 μM) and 5-AZA (5 μM) group varied considerably compared to those from the control group. The grey values of DNMT1, p53, and p21 in the Western blots were analyzed for estimating the protein amount (**Figure 4B**).

### CGA Inhibits the Activation of ERK1/2 and MMP2/9 in HepG2 Cells

The mitogen-activated protein kinases (MAPK) signaling pathway is critical to cell proliferation. Immunoblotting results confirmed ERK1/2 activation based on the phosphorylated ERK1/2 level. After 48 h of treatment, CGA suppressed ERK1/2 phosphorylation and MMP2/9 expression in HepG2 cells (**Figures 4C, D**).

### CGA Decreased DNMT1 Levels in HepG2 Cells by Immunocytochemistry Staining

**Figure 5:**
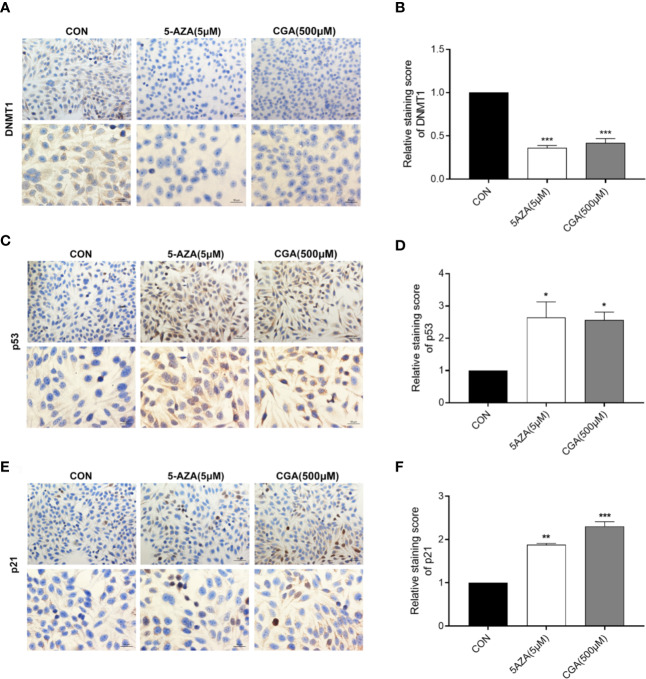
Immunocytochemical staining intensity after treatment with CGA (250, 500, 1000 μM) and 5-AZA (5 μM) (200 × and 400 × magnification). **(A–F)** DAB staining showed a considerable decrease in DNMT1 expression and an increase in p53 and p21 expression in the CGA group. *p < 0.05, **p < 0.01, ***p < 0.001. CON, vehicle DMSO control group.

**Figure 5** shows positive immunocytochemical staining of DNMT1, p53, and p21 in the cytoplasm of HepG2 cells. DNMT1 and p53 showed diffuse staining, whereas p21 showed a granular staining pattern. CGA pretreated cells showed a significantly lower DNMT1 immunoreactivity than control cells, and similar results were observed with 5-AZA treated cells (**Figure 5A**). CGA pretreated cells showed a considerably stronger immunoreactivity toward p53 and p21 than control cells (**Figures 5C, E**). The intensity of the immunocytochemical staining of DNMT1, p53, and p21 was analyzed in addition to protein levels, and significant differences were observed in case of cells from the CGA (500 μM), and 5-AZA (5 μM) groups, compared to those from the control group (**Figures 5B, D, F**). The results showed that in comparison to the control group, the DNMT1 protein levels were considerably lower in the CGA group. In contrast, in cells treated with CGA, the p53 and p21 levels were markedly higher than those in the cells from the control group. These results showed that decrease in DNMT1 expression inhibits HCC cell proliferation by up-regulating p53 and p21.

### Anticancer Effect of CGA Against HepG2 Xenografts

**Figure 6:**
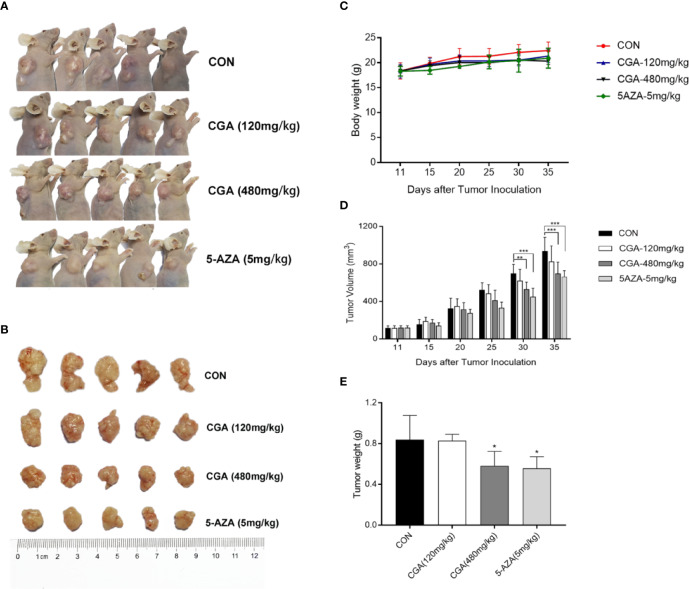
CGA reduces HepG2 tumor xenograft’s weight and volume. HepG2 cells were transplanted into nude mice and divided randomly into four groups: CON, CGA (120 and 480 mg/kg), and 5-AZA (5 mg/kg), and treated every day for 24 days from 11 days after tumor inoculation **(A, B)**. **(C)** Mouse body weights were measured every five days, from day 11 to day 35. **(D)** HepG2 xenograft tumor progression was assessed by tumor volume measurement every five days. **(E)** After 35 days, the tumor was resected, and weights measured. *p < 0.05, **p < 0.01, ***p < 0.001. CON, control group receiving intraperitoneal injection of normal saline.

The *in vivo* anticancer effect of CGA was evaluated in immunodeficient HepG2 xenograft nude mouse models. The mice were divided randomly into four groups: Group 1, injected with saline, groups 2 and 3, injected with CGA at 120 and 480 mg/kg, respectively, and Group 4, injected with 5-AZA 5 mg/kg every day from the first day of tumor formation. Compared to the control (Group 1), treatment with CGA at 120 mg/kg (Group 2), 480 mg/kg (group 3), and 5-AZA at 5 mg/kg (Group 4) inhibited tumor growth, especially in case of 480 mg/kg CGA and 5 mg/kg 5-AZA (**Figures 6A, B**). All the mice were healthy. Slight differences were observed in body weight among the four groups (**Figure 6C**). Tumor volumes of mice xenografts in groups 3 and 4 were reduced considerably by 18.5% and 28.0%, respectively, compared to the control (Group 1) (**Figure 6D**), while the weights of the xenograft tumors in mice from groups 3 and 4 were reduced by 30.7% and 33.4%, respectively, compared to those in case of mice from the control (Group 1) (**Figure 6E**). These results showed that CGA inhibits the *in vivo* tumor growth in HepG2 xenograft mice.

**Figure 7:**
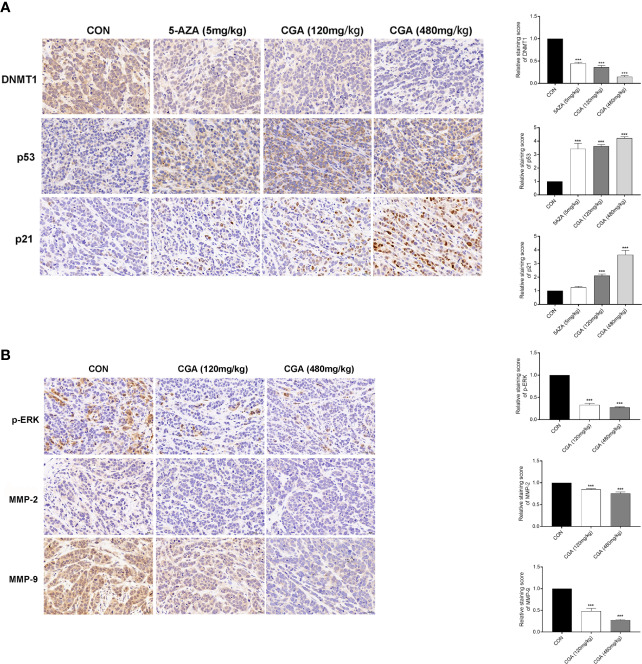
Detection of DNMT1, p53, p21, p-ERK, MMP-2, and MMP-9 expression in HepG2 xenografts by immunohistochemistry (400× magnification). **(A)** Compared with the CON group, DAB staining showed a considerable decrease in DNMT1 expression and an increase in p53 and p21 expression in the CGA group. **(B)** And a considerable decrease in p-ERK, MMP-2 and MMP-9 expression in the CGA group. ***p < 0.001. CON, control group receiving intraperitoneal injection of normal saline.

Additionally, the protein levels of DNMT1, p53, p21, p-ERK, MMP-2, and MMP-9 in tumors were assessed by immunohistochemical analysis, and the results showed that compared to the control group, the levels of these proteins in the CGA and 5-AZA groups were reduced (**Figure 7A**). The *in vivo* results showed an apparent decrease in the levels of DNMT1 after treatment with CGA or 5-AZA, and this effect was higher with increasing CGA concentrations. By maintaining the stability and localization of p53 and p21, a low level of DNMT1 inhibits the formation and growth of the HCC xenograft tumor. Moreover, CGA inhibits the HepG2 xenograft growth by inactivating ERK and decreasing MMP-2/9, thus inhibiting extracellular matrix degradation and HepG2 xenograft development (**Figure 7B**).

## Discussion

HCC treatment methods should explore effective chemoprevention strategies. Epidemiological studies showed that CGA ameliorates certain chronic diseases and cancers (Feng et al., 2005; Wang et al., 2011; Intisar et al., 2012). However, the CGA-affected DNMT1 expression-mediated mechanism is not yet understood.

In this study, we used the HCC cell line HepG2, and HepG2 nude mice xenografts, to evaluate inhibition of HCC growth both *in vivo* and *in vitro*. At higher concentrations, CGA reduced *in vitro* HepG2 cell viability, colony formation, invasion and migration (**Figures 2** and **3**), and *in vivo*, CGA treatment inhibited growth of HepG2 xenograft tumors, at both 120 and 480 mg/kg of CGA causing reduction in tumor weight and tumor volume (**Figure 6**).

At concentrations higher than 250 μM, CGA showed a significant inhibitory effect, and at a concentration of 1000 μM, it caused considerable S-phase arrest in HepG2 cells, which was consistent with other studies (Yan et al., 2017).

The regulation of genes involved in epigenetic processes is critical to understanding cancer development. DNA methylation is an essential epigenetic event in maintaining cellular function and regulating gene expression, probably facilitating cancer development (Butt et al., 2009; Lai et al., 2012; Bayan et al., 2014). DNMT1 is a maintenance methylase, critical throughout DNA methylation (Dan and Chen, 2016). The p53 protein is a tumor repressed gene, involved in regulating uncontrolled cell division, DNA replication, and cell cycle during tumor growth(Luo et al., 2017). When p53 protein loses its regulatory function, it may cause tumor progression and growth. Cyclin-dependent kinase (CDK) inhibitors (e.g., the cip/Kip family proteins, such as p21waf/cip1) bind to, and inhibit the activity of cyclin-CDK complexes, thereby participating as a negative regulator of cell cycle progression (Elledge et al., 1996; Harper and Elledge, 1996). In addition, a previous study indicated that DNMT1 could translocate into the nucleus, inhibiting p53 transcription and leading to breast cancer progression by the activation of autophagy (Du et al., 2018). The inhibition of p53 expression was relieved in the absence of DNMT1, leading to the apoptosis of pancreatic progenitor cells (Georgia et al., 2013). Long noncoding RNA ATB could accelerate the proliferative and migratory rates of renal cell carcinoma cells and inhibit cell apoptosis through downregulation of p53 *via* binding to DNMT1 (Song et al., 2019). In this study, we showed a link between DNMT1 and p53, p21. The Western blotting analysis showed that CGA treatment resulted in decreased DNMT1 levels and increased p53 and p21 levels in HepG2 cells (**Figure 4**). This study reports for the first time, the effect of CGA on the expression of DNMT1 in HCC. Protein expression in HepG2 cells examined by immunocytochemical methods showed a significant decrease in DNMT1 expression and increased p53, p21 in the CGA group (**Figure 5**). The above data showed the variable effect of CGA on the levels of DNMT1, p53, and p21 in HepG2 cells, leading to suppression of HCC proliferation.

ERK abnormalities participate in HCC cell proliferation processes and tumor progression (Shimizu et al., 2015). MMPs are critical for tumor invasion and metastasis by degenerating matrix proteins located on or outside the cells (e.g., proteoglycans and collagens) (Fassina et al., 2000; Mannello et al., 2005). Our results also showed that CGA inhibited HepG2 cell proliferation and HCC growth by inactivation of ERK and down-regulation of MMP-2/9, which prevented the degradation of the extracellular matrix (**Figure 4**); thus, these mechanisms may be involved in its anti-tumor effect.

## Data Availability Statement

The datasets generated for this study are available on request to the corresponding authors.

## Ethics Statement

The animal study was reviewed and approved by Vital River Institutional Animal Care and Use Committee.

## Author Contributions

YF and XW designed this study and supervised the entire process. YaL performed IHC, carried out the majority of the vitro experiments, and performed cell viability and migration/invasion assay. GZ supervised the vivo experiments. YuL conducted the majority of the vivo experiments. YiH reviewed IHC slides. YaL wrote the manuscript. All authors contributed to the article and approved the submitted version.

## Funding

This study was supported by the Beijing Municipal Administration of Hospitals clinical medicine development of special funding support (No. ZYLX201707), the Capital’s Funds for Health Improvement and Research (CFH2018-1-2172), the Beijing Municipal Natural Science Foundation (No. 7184219) and the Beijing Outstanding Talent Training Project (No. 2017000021469G297).

## Conflict of Interest

The authors declare that the research was conducted in the absence of any commercial or financial relationships that could be construed as a potential conflict of interest.
